# Cotrimoxazole prophylaxis and antiretroviral therapy: an observational cohort study in China

**DOI:** 10.2471/BLT.14.142745

**Published:** 2015-01-29

**Authors:** Wei Cheng, Yasong Wu, Yi Wen, Ye Ma, Decai Zhao, Zhihui Dou, Weiwei Zhang, Marc Bulterys, Fujie Zhang

**Affiliations:** a Division of Treatment and Care, National Center for AIDS/STD Control and Prevention, Chinese Center for Disease Control and Prevention, 27 Nanwei Rd, Beijing 100050, China.; b Global AIDS Program, Centers for Disease Control and Prevention, United States Embassy, Beijing, China.

## Abstract

**Objective:**

To assess if cotrimoxazole prophylaxis administered early during antiretroviral therapy (ART) reduces mortality in Chinese adults who are infected with human immunodeficiency virus (HIV).

**Methods:**

We did a retrospective observational cohort study using data from the Chinese national free antiretroviral database. Patients older than 14 years who started ART between 1 January 2010 and 31 December 2012 and had baseline CD4+ T-lymphocyte (CD4+ cell) count less than 200 cells/µL were followed until death, loss to follow-up or 31 December 2013. Hazard ratios (HRs) for several variables were calculated using multivariate analyses.

**Findings:**

The analysis involved 23 816 HIV-infected patients, 2706 of whom died during the follow-up. Mortality in patients who did and did not start cotrimoxazole during the first 6 months of ART was 5.3 and 7.0 per 100 person–years, respectively. Cotrimoxazole was associated with a 37% reduction in mortality (hazard ratio, HR: 0.63; 95% confidence interval, CI: 0.56–0.70). Cotrimoxazole in addition to ART reduced mortality significantly over follow-up lasting 6 months (HR: 0.65; 95% CI: 0.59–0.73), 12 months (HR: 0.58; 95% CI: 0.49–0.70), 18 months (HR: 0.49; 95% CI: 0.38–0.63) and 24 months (HR: 0.66; 95% CI: 0.48–0.90). The mortality reduction was evident in patients with baseline CD4+ cell counts less than 50 cells/µL (HR: 0.60; 95% CI: 0.54–0.67), 50–99 cells/µL (HR: 0.66; 95% CI: 0.56–0.78) and 100–199 cells/µL (HR: 0.78; 95% CI: 0.62–0.98).

**Conclusion:**

Cotrimoxazole prophylaxis started early during ART reduced mortality and should be offered to HIV-infected patients in low- and middle-income countries.

## Résumé

**Objectif:**

Évaluer si la prophylaxie par le cotrimoxazole administrée précocement au cours de la thérapie antirétrovirale (TAR) réduit la mortalité chez les adultes infectés par le virus de l'immunodéficience humaine (VIH) en Chine.

**Méthodes:**

Nous avons effectué une étude de cohorte par observation rétrospective en utilisant les données de la base de données antirétrovirale gratuite nationale chinoise. Les patients âgés de plus de 14 ans qui ont commencé une TAR entre le 1er janvier 2010 et le 31 décembre 2012 et qui avaient un nombre de lymphocytes T CD4+ (cellules CD4+) à la ligne de base inférieur à 200 cellules/µL, ont été suivis jusqu'à leur décès, jusqu'à ce qu'ils soient perdus de vue au suivi ou jusqu'au 31 décembre 2013. Les rapports des risques (RR) pour plusieurs variables sont calculés en utilisant des analyses multivariées.

**Résultats:**

Le suivi. La mortalité chez les patients qui ont et qui n'ont pas commencé à prendre le cotrimoxazole au cours des 6 premiers mois de la TAR était de 5,3 et 7,0 pour 100 personnes-années, respectivement. Le cotrimoxazole était associé avec 37% de réduction de la mortalité (rapport des risques, RR: 0,63; intervalle de confiance à 95%, IC 95%: 0,56-0,70). Le cotrimoxazole ajouté à la TAR a réduit significativement la mortalité au cours du suivi des 6 derniers mois (RR: 0,65; IC 95%: 0,59–0,73), des 12 derniers mois (RR: 0,58; IC 95%: 0,49-0,70), des 18 derniers mois (RR: 0,49; IC 95%: 0,38-0,63) et des 24 derniers mois (RR: 0,66; IC 95%: 0,48-0,90). La réduction de la mortalité était évidente chez les patients avec un nombre de cellules CD4+ à la ligne de base inférieur à 50 cellules/µL (RR: 0,60; IC 95%: 0,54–0,67), 50–99 cellules/µL (RR: 0,66; IC 95%: 0,56-0,78) et 100-199 cellules/µL (RR: 0,78; IC 95%: 0,62-0,98).

**Conclusion:**

La prophylaxie par le cotrimoxazole commencée précocement au cours de la TAR a réduit la mortalité et devrait être proposée aux patients infectés par le VIH dans les pays à revenu faible et à revenu intermédiaire.

## Resumen

**Objetivo:**

Evaluar si la profilaxis con cotrimoxazol en una etapa temprana de la terapia antirretroviral (TAR) reduce la mortalidad en adultos de origen chino infectados por el virus de la inmunodeficiencia humana (VIH).

**Métodos:**

Se llevó a cabo un estudio de cohorte observacional retrospectivo utilizando los datos de la base de datos nacional de China de antirretrovirales de uso gratuito. Por otro lado, se realizó un seguimiento a pacientes mayores de 14 años que iniciaron el tratamiento con TAR entre el 1 de enero de 2010 y el 31 de diciembre de 2012 y con recuento basal de linfocitos T CD4+ (célula CD4+) inferior a 200 células/µl hasta su muerte, la pérdida de seguimiento o el 31 de diciembre de 2013. Se calcularon los cocientes de riesgos (CR) para diversas variables mediante análisis multivariados.

**Resultados:**

En el análisis participaron 23 816 pacientes infectados por el VIH, 2706 de los cuales fallecieron durante el seguimiento. La mortalidad entre los pacientes que iniciaron y no iniciaron el tratamiento con cotrimoxazol durante los primeros 6 meses de la TAR fue de 5,3 y 7,0 por 100 personas/años, respectivamente. Cotrimoxazol se asoció a una reducción del 37 % en la mortalidad (cociente de riesgos, CR: 0,63, intervalo de confianza del 95 %, IC: 0,56 – 0,70). Además de la TAR, cotrimoxazol redujo la mortalidad considerablemente durante el seguimiento de 6 meses (CR: 0,65; IC del 95 %: 0,59 – 0,73), 12 meses (CR: 0,58; IC del 95 %: 0,49 – 0,70), 18 meses (CR: 0,49; IC del 95 %: 0,38 – 0,63) y 24 meses (CR: 0,66; IC del 95 %: 0,48 – 0,90). La reducción de la mortalidad resultó evidente en pacientes con recuento basal de células CD4+ inferior a 50 células/µl (CR: 0,60; IC del 95 %: 0,54 – 0,67), 50 – 99 células/µl (CR: 0,66; IC del 95 %: 0,56 – 0,78) y 100 – 199 células/µl (CR: 0,78; IC del 95 %: 0,62 – 0,98).

**Conclusión:**

La profilaxis con cotrimoxazol iniciada en una etapa temprana de la TAR redujo la mortalidad y debe ofrecerse a los pacientes infectados por el VIH en países de ingresos bajos y medios.

## ملخص

**الغرض:**

تقييم ما إذا كانت الوقاية باستخدام الكوتريموكسازول في مرحلة مبكرة خلال العلاج بمضادات الفيروسات القهقرية يقلل معدل الوفيات بين البالغين المصابين بفيروس العوز المناعي البشري في الصين.

**الطريقة:**

أجرينا دراسة أترابية استرجاعية قائمة على الملاحظة باستخدام البيانات المستمدة من قاعدة بيانات مضادات الفيروسات القهقرية المجانية الوطنية في الصين. وتم متابعة المرضى الأكبر من 14 عاماً الذين استهلوا العلاج بمضادات الفيروسات القهقرية في الفترة من 1 كانون الثاني/ يناير 2010 حتى 31 كانون الأول/ ديسمبر 2012 وكان إحصاء الخلايا اللمفاوية التائية المساعدة (CD4+) عند خط الأساس لديهم أقل من 200 خلية/ميكرو لتر حتى الوفاة، أو فقدان المتابعة، أو حتى 31 كانون الأول/ ديسمبر 2013. وتم حساب نسب المخاطر لعدة متغيرات باستخدام التحليلات متعددة المتغيرات.

**النتائج:**

اشتمل التحليل على 23816 مريضاً مصاباً بفيروس العوز المناعي البشري، توفي منهم 2706 مريضاً أثناء المتابعة. وكان معدل الوفيات بين المرضى الذين استهلوا ولم يستهلوا استخدام الكوتريموكسازول خلال الستة أشهر الأولى من العلاج بمضادات الفيروسات القهقرية 5.3 و7.0 لكل 100 شخص إلى عدد السنوات، على التوالي. وارتبط الكوتريموكسازول بانخفاض في معدل الوفيات نسبته 37 % (نسبة المخاطر: 0.63؛ فاصل الثقة 95 %، فاصل الثقة: من0.56 إلى 0.70). وأدى استخدام الكوتريموكسازول بالإضافة إلى العلاج بمضادات الفيروسات القهقرية إلى تقليل معدل الوفيات بشكل كبير على مدار فترة المتابعة التي دامت 6 أشهر (نسبة المخاطر: 0.65؛ فاصل الثقة 95 %: من 0.59 إلى 0.73)، و12 شهراً (نسبة المخاطر: 0.58؛ فاصل الثقة 95 %: من 0.49 إلى 0.70)، و18 شهراً (نسبة المخاطر: 0.49؛ فاصل الثقة 95 %: من 0.38 إلى 0.63)، و24 شهراً (نسبة المخاطر: 0.66؛ فاصل الثقة 95 %: من 0.48 إلى 0.90). واتضح الانخفاض في معدل الوفيات بين المرضى الذين كانت إحصاءات الخلايا اللمفاوية التائية المساعدة (CD4+) عند خط الأساس لديهم أقل من 50 خلية/ميكرو لتر (نسبة المخاطر: 0.60؛ فاصل الثقة 95 %: من 0.54 إلى 0.67)، ومن 50 إلى 99 خلية/ميكرو لتر (نسبة المخاطر: 0.66؛ فاصل الثقة 95 %: من 0.56 إلى 0.78)، ومن 100 إلى 199 خلية/ميكرو لتر (نسبة المخاطر: 0.78؛ فاصل الثقة 95 %: من 0.62 إلى 0.98).

**الاستنتاج:**

أدت الوقاية باستخدام الكوتريموكسازول التي تم بدأت في مرحلة مبكرة خلال العلاج بمضادات الفيروسات القهقرية إلى تقليل معدل الوفيات ومن ثم ينبغي توفيرها للمرضى المصابين بفيروس العوز المناعي البشري في البلدان المنخفضة الدخل والبلدان المتوسطة الدخل.

## 摘要

**目的:**

评估抗逆转录病毒治疗（ART）早期进行磺胺甲基异恶唑预防疗法是否降低中国艾滋病病毒（HIV）感染成年人的死亡率。

**方法:**

我们使用中国国家免费抗逆转录病毒数据库的数据进行回顾性观察性队列研究。对14岁以上在2010年1月1日至2012年12月31日之间开始ART治疗并且基准CD4+T淋巴细胞（CD4+细胞）计数小200 cells/µL的患者进行跟踪随访，直至死亡、失访或到2013年12月31日。使用多元分析计算数个变量的风险比（HR）。

**结果:**

该分析涉及23816名艾滋病病毒感染者，其中2706人在随访期间死亡。在ART治疗前6个月开始接受或者未接受磺胺甲基异恶唑预防疗法的病人死亡率分别为每100人年5.3和7.0。磺胺甲基异恶唑预防疗法与死亡率降低37%（危害比、HR：0.63；95%置信区间，CI：0.56-0.70）相关。ART结合磺胺甲基异恶唑预防疗法显著降低持续6个月（HR：0.65；95% CI：0.59-0.73）、12个月（HR：0.58；95% CI：0.49-0.70）、18个月（HR：0.49；95% CI：0.38-0.63）和24个月（HR：0.66；95% CI：0.48-0.90）随访期间的死亡率。基准CD4+细胞计数小于50 cells/µL（HR：0.60；95% CI：0.54-0.67）、50-99 cells/µL（HR：0.66；95% CI：0.56-0.78）和100-199 cells/µL（HR：0.78；95% CI：0.62-0.98）的患者的死亡率降低明显。

**结论:**

在ART治疗早期加入磺胺甲基异恶唑预防疗法可降低死亡率，应为中低收入国家艾滋病病毒感染者提供此疗法。

## Резюме

**Цель:**

Определить, приводит ли профилактика котримоксазолом на раннем этапе антиретровирусной терапии (АРТ) к снижению смертности среди ВИЧ-инфицированных взрослых в Китае.

**Методы:**

Было проведено ретроспективное обсервационное когортное исследование с использованием данных Китайской национальной базы данных свободного доступа по антиретровирусной терапии. Пациенты старше 14 лет, которые начали получать АРТ в период с 1 января 2010 года по 31 декабря 2012 года, и исходное число CD4+ T-лимфоцитов которых составляло менее 200 клеток/мкл, находились под наблюдением до летального исхода, выбытия из наблюдения или до 31 декабря 2013 года. С помощью многомерных анализов определялось отношение рисков (ОР) для нескольких переменных.

**Результаты:**

Анализ охватывал 23 816 ВИЧ-инфицированных пациентов, 2706 из которых скончались во время наблюдения. Уровень смертности среди пациентов, начавших и не начавших прием котримоксазола в течение первых 6 месяцев АРТ, составлял 5,3 и 7,0 на 100 человеко-лет соответственно. Котримоксазол ассоциировался с 37%‑ным снижением уровня смертности (отношение рисков (ОР): 0,63; 95%‑ный доверительный интервал (ДИ): 0,56-0,70). Котримоксазол в дополнение к АРТ значительно снижал смертность во время последующего наблюдения, длившегося 6 месяцев (ОР: 0,65; 95% ДИ: 0,59–0,73), 12 месяцев (ОР: 0,58; 95% ДИ: 0,49-0,70), 18 месяцев (ОР: 0,49; 95% ДИ: 0,38-0,63) и 24 месяца (ОР: 0,66; 95% ДИ: 0,48-0,90). Заметное снижение смертности наблюдалось у пациентов с исходным показателем CD4+ менее 50 клеток/мкл (ОР: 0,60; 95% ДИ: 0,54–0,67), 50–99 клеток/мкл (ОР: 0,66; 95% ДИ: 0,56–0,78) и 100–199 клеток/мкл (ОР: 0,78; 95% ДИ: 0,62-0,98).

**Вывод:**

Профилактика котримоксазолом, начатая на раннем этапе АРТ, приводила к снижению смертности и должна предлагаться ВИЧ-инфицированным пациентам в странах с низким и средним уровнями доходов.

## Introduction

With the rapid rise in the availability of antiretroviral therapy (ART), the number of people dying from acquired immune deficiency syndrome (AIDS) has dramatically declined. However, in many low- and middle-income countries, ART coverage remains relatively poor.^1^ Moreover, mortality during the early stages of ART is higher in low- than high-income countries, even after adjusting for immunodeficiency at baseline.^2^ Reducing AIDS-related mortality in low- and middle-income countries will require the continued expansion of ART coverage, improved regimens and the adoption of additional cost-effective interventions.

Prophylaxis with cotrimoxazole – a combination of the antibiotics trimethoprim and sulfamethoxazole – provides protection against several opportunistic infections, including *Pneumocystis jirovecii* pneumonia, malaria and cerebral toxoplasmosis.^3^^–^^6^ Many studies have demonstrated that cotrimoxazole prophylaxis increases survival among ART-naïve patients: reductions in mortality of 19 to 46% have been reported in low- and middle-income countries.^6^^–^^12^ The World Health Organization (WHO) recommends that cotrimoxazole be given in such settings to adolescents (10–19 years) and adults with advanced human immunodeficiency virus (HIV) clinical disease or a CD4+ T lymphocyte (CD4+) cell count less than 350 cells/µL.^13^ Several studies have reported that cotrimoxazole provides a continuous protective effect after ART initiation.^14^^–^^20^ Yet, the implementation of cotrimoxazole prophylaxis remains a challenge in low- and middle-income settings because of weak drug procurement systems, problems with managing the supply chain and little awareness of this recommendation among medical staff.^19^^–^^21^ The situation is similar in China where some health-care workers are still unaware of the substantial benefits of cotrimoxazole prophylaxis in eligible HIV-infected patients.

In 2003, China officially launched its national free antiretroviral treatment programme. Two years later, the first guidelines on cotrimoxazole in the country were released. They recommended that cotrimoxazole prophylaxis be given to all individuals older than 14 years with a CD4+ cell count less than 200 cells/µL, WHO stage-4 disease or a history of oropharyngeal candidiasis. Several studies have reported substantially better survival among HIV-infected patients since the programme was launched.^22^^–^^24^ However, nationally-representative data on the implementation of cotrimoxazole prophylaxis has been available only since 2010, when these first started to be recorded in the programme’s information system. Consequently, although the protective effect of cotrimoxazole has been documented elsewhere, evidence from China has been limited. The aim of this study was to use data from the programme’s database to evaluate if cotrimoxazole prophylaxis initiated early during ART was associated with reduced mortality among HIV-infected patients.

## Methods

We performed a retrospective, observational, cohort study using data from the national free antiretroviral treatment programme database, held at the Chinese Center for Disease Control and Prevention. The database contains information on the treatment of all Chinese patients with HIV infections, who are managed by the programme. In accordance with national policy, local health-care providers recorded details of treatment at initiation, at follow-up visits 0.5, 1, 2 and 3 months later and once every 3 months thereafter. If the regimen changes, the follow-up schedule is restarted at 0.5 months. As in previous analyses,^24^^,^^25^ patients were considered lost to follow-up if they missed four consecutive follow-up appointments and their records were terminated at the date of the last follow-up visit. Hard copies of all treatment data were kept at the treatment sites and all information was entered into the programme’s database.^25^ By 31 December 2013, the database contained information on 278 085 HIV-infected patients.

**Fig. 1:**
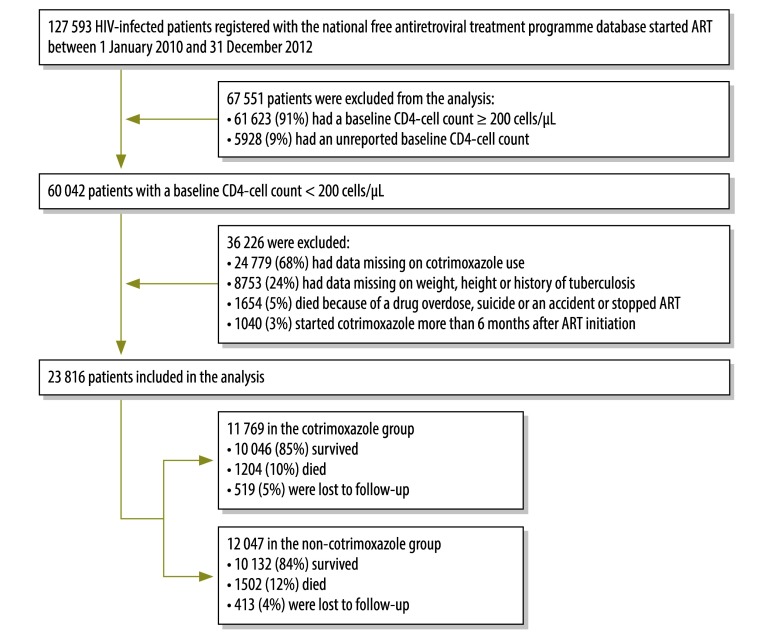
Flowchart for the selection of HIV-infected patients who received antiretroviral therapy, China, 2010–2013

ART: antiretroviral therapy; HIV: human immunodeficiency virus.

Although cotrimoxazole has been officially recommended in China since 2005, the national free antiretroviral treatment programme database did not record cotrimoxazole use until 1 January 2010, when data collection was changed from a fax-based system to an internet-based system. Records were screened and selected from among those in the programme database on 1 January 2014 if the following inclusion criteria were satisfied: (i) ART was initiated between 1 January 2010 and 31 December 2012; (ii) the patient’s baseline CD4+ cell count was less than 200/µL; (iii) there was a record of whether or not the patient received cotrimoxazole during every scheduled follow-up visit; and (iv) details of the patient’s weight, height and history of tuberculosis at baseline were available. We excluded records of patients who died from either a drug overdose, suicide or an accident, who started cotrimoxazole more than 6 months after ART initiation or who stopped ART (Fig. 1).

### Cotrimoxazole prophylaxis

Cotrimoxazole was administered orally and stopped in accordance with instructions in the *China free ART manual*.^26^ Since the database recorded only information on whether or not the patient was receiving cotrimoxazole at each follow-up visit and did not register the exact date on which the drug was started or stopped, we regarded any patient who reported cotrimoxazole use at one or more scheduled follow-up visits within 6 months of ART initiation as belonging to our cotrimoxazole group. Our non-cotrimoxazole group comprised patients who did not report cotrimoxazole use at any follow-up visit following ART initiation.

The observation period in the non-cotrimoxazole group was the time from the date of ART initiation to the date of death, loss to follow-up or the last follow-up visit before 31 December 2013. For the cotrimoxazole group, the observation period was the time from the date of the last follow-up visit before the visit during which cotrimoxazole use was first reported to the date of death, loss to follow-up or the last follow-up visit before 31 December 2013. For example, if cotrimoxazole prophylaxis was first reported in the fourth follow-up visit, the observation period started at the date of the third follow-up visit. Since we did not know exactly when cotrimoxazole was stopped, we assumed that patients in the cotrimoxazole group continued to take the drug throughout the observation period and, therefore, regarded the duration of cotrimoxazole prophylaxis as being identical to the observation period. The study was approved by the institutional review board of the National Center for AIDS/STD (acquired immunodeficiency syndrome/sexually transmitted diseases) Control and Prevention.

### Statistical analysis

Differences between the groups in baseline characteristics, such as age, sex, marital status, HIV infection route, baseline CD4+ cell count, WHO clinical stage and tuberculosis infection in the year before ART initiation, were compared using an *χ*^2^ test for categorical variables and a Mann–Whitney *U* test for continuous variables since no continuous variable fulfilled the Kolmogorov–Smirnov test for normality. A low body mass index (BMI) was defined as a value less than 18.5 kg/m^2^, in accordance with the threshold for malnutrition of the Food and Agriculture Organization of the United Nations.^27^ Patients’ data were censored at 31 December 2013 or the date of loss to follow-up or death, depending on which was earliest. The association between mortality and cotrimoxazole and baseline CD4+ cell count after ART initiation was analysed using Kaplan–Meier survival curves and crude mortality rates. In addition, mortality was also evaluated using Cox proportional hazards regression models that included adjustment for covariates that were predetermined to be clinically meaningful and which had a significant influence in the unadjusted analysis (i.e. a *P*-value < 0.1). To determine whether cotrimoxazole’s influence on mortality varied according to the duration of ART and the baseline CD4+ cell count, the Cox models were stratified by the duration of ART (i.e. 6, 12, 18, 24 and 30 months) and the baseline CD4+ cell count (i.e. < 50, 50–99 and 100–199 cells/µL). Finally, to confirm our findings, we introduced a category for missing data and repeated the calculations using data that included observations from individuals who had been excluded because of missing records. All calculations were performed using SAS version 9.2 (SAS Institute, Cary, United States of America).

## Results

The analysis included data on 23 816 patients aged 15 years or more: 12 047 (51%) had never taken cotrimoxazole, whereas 11 769 (49%) had taken the drug within 6 months of ART initiation (Fig. 1). Of those who reported cotrimoxazole use, 2252 (19%) did not begin taking the drug at ART initiation: the mean starting time was 27 days (interquartile range, IQR: 14–56) after ART initiation. The median age of all participants was 40 years (IQR: 33–49), 71% (16 976) were male and the most common route of HIV infection was sexual transmission, followed by injection-drug use, blood or plasma transfusion and other or unknown routes. The patients’ median baseline CD4+ cell count was 84 cells/µL – the median was significantly lower in the cotrimoxazole group, at 69 cells/µL, compared with 103 cells/µL in the non-cotrimoxazole group (Table 1). In addition, significantly more patients in the cotrimoxazole group than the non-cotrimoxazole group had a low BMI (28% versus 21%, respectively), a history of tuberculosis before ART (15% versus 10%, respectively) and WHO clinical stage-3 disease (28% versus 22%, respectively) or stage-4 disease (31% versus 18%, respectively).

**Table 1:** Characteristics of HIV-infected patients receiving antiretroviral therapy, by cotrimoxazole prophylaxis, China, 2010–2013

Characteristic	No. (%)^a^ of patients (*n* = 23 816)	No. (%)^a^ of patients in non-cotrimoxazole group^b ^(*n* = 12 047)	No. (%)^a^ of patients in cotrimoxazole group^c^ (*n* = 11 769)	*P*
**Duration of follow-up in months, median (IQR)**	21 (14–30)	20 (14–29)	23 (15–32)	< 0.001
**Number of scheduled follow-up visits, median (IQR)**	12 (8–16)	11 (8–15)	12 (9–17)	< 0.001
**Age in years, median (IQR)**	40 (33–49)	40 (32–49)	41 (33–50)	0.009
**Age, years^d^**				**< 0.001**
< 30	3 730 (16)	2 089 (17)	1 641 (14)	ND
30–39	7 621 (32)	3 774 (31)	3 847 (33)	ND
40–49	6 562 (28)	3 261 (27)	3 301 (28)	ND
50–59	3 251 (14)	1 594 (13)	1 657 (14)	ND
≥ 60	2 652 (11)	1 329 (11)	1 323 (11)	ND
**Sex**				< 0.001
Male	16 976 (71)	8 744 (73)	8 232 (70)	ND
Female	6 840 (29)	3 303 (27)	3 537 (30)	ND
**Marital status**				< 0.001
Married or living with partner	15 021 (63)	7 301 (61)	7 720 (66)	ND
Single, divorced or widowed	8 795 (37)	4 746 (39)	4 049 (34)	ND
**Route of HIV transmission^d^**				< 0.001
Sexual transmission	19 105 (80)	9 919 (82)	9 186 (78)	ND
Injection-drug use	2 221 (9)	982 (8)	1 239 (11)	ND
Blood or plasma transfusion	1 405 (6)	601 (5)	804 (7)	ND
Other or unknown	1 085 (5)	545 (5)	540 (5)	ND
**Baseline CD4+ cell count in cells/µL, median (IQR)**	84 (31–148)	103 (39–159)	69 (25–133)	< 0.001
**Baseline CD4+ cell count, cells/µL**				< 0.001
< 50	8 357 (35)	3 576 (30)	4 781 (41)	ND
50–99	4 931 (21)	2 308 (19)	2 623 (22)	ND
100–199	10 528 (44)	6 163 (51)	4 365 (37)	ND
**BMI in kg/m^2^, median (IQR)**	20 (19–22)	21 (19–22)	20 (18–22)	< 0.001
**Body mass index, kg/m^2^**				< 0.001
≥ 18.5	17 947 (75)	9 513 (79)	8 434 (72)	ND
< 18.5	5 869 (25)	2 534 (21)	3 335 (28)	ND
**WHO clinical stage^d^**				< 0.001
1	7 212 (30)	4 376 (36)	2 836 (24)	ND
2	4 764 (20)	2 753 (23)	2 011 (17)	ND
3	6 014 (25)	2 699 (22)	3 315 (28)	ND
4	5 826 (24)	2 219 (18)	3 607 (31)	ND
**Tuberculosis before ART**				< 0.001
Yes	2 930 (12)	1 161 (10)	1 769 (15)	ND
No	20 886 (88)	10 886 (90)	10 000 (85)	ND
**Cotrimoxazole started at ART initiation**				NA
Yes	NA	NA	9 517 (81)	NA
No	NA	NA	2 252 (19)	NA

ART: antiretroviral therapy; BMI: body mass index; HIV: human immunodeficiency virus; IQR: interquartile range; NA: not applicable; ND: not determined; WHO: World Health Organization.

^a^ All values in the table represent absolute numbers and percentages unless otherwise stated.

^b^ The non-cotrimoxazole group comprised HIV-infected patients who did not report cotrimoxazole use at any time after starting antiretroviral therapy.

^c^ The cotrimoxazole group comprised HIV-infected patients who reported cotrimoxazole use within 6 months of starting antiretroviral therapy.

^d^ Percentages may not sum to 100% due to rounding.

**Fig. 2:**
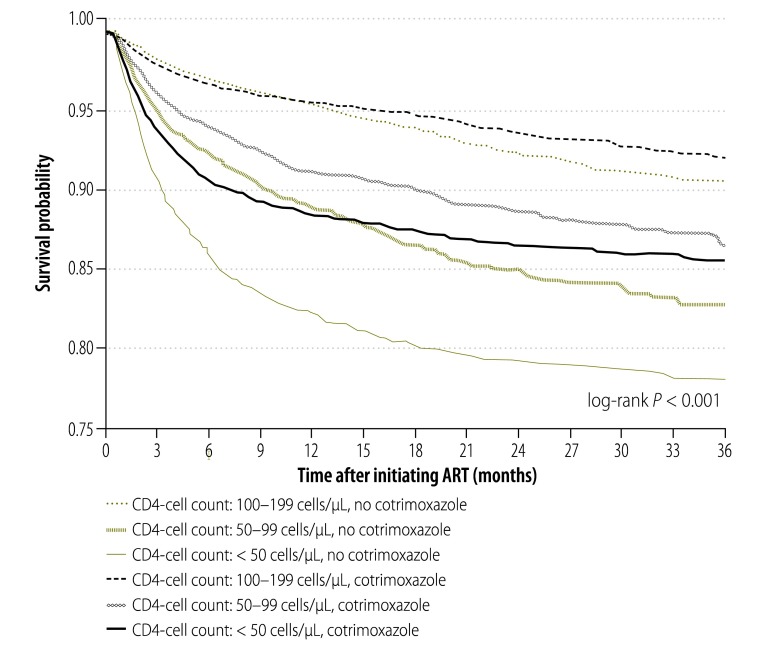
Kaplan–Meier survival curves for HIV-infected patients receiving antiretroviral therapy, by cotrimoxazole use and baseline CD4+ cell count, China, 2010–2013

ART: antiretroviral therapy; HIV: human immunodeficiency virus.

Note: The *P*-value indicates the significant survival rate by cotrimoxazole use and baseline CD4+ cell count.

In total, 2706 patients died during 44 137 person–years of observation: 56% (1515/2706) were in the non-cotrimoxazole group and 44% (1191/2706) were in the cotrimoxazole group. Overall mortality was 6.1 per 100 person–years: 7.0 per 100 person-years in the non-cotrimoxazole group and 5.3 per 100 person–years in the cotrimoxazole group. Kaplan–Meier survival curves showed that survival was lowest in individuals with a baseline CD4+ cell count less than 50 cells/µL who did not receive cotrimoxazole (Fig. 2).

Univariate analysis found that the risk of death was significantly lower in patients who received cotrimoxazole (hazard ratio, HR: 0.80), who were female (HR: 0.75) and who had no history of tuberculosis before ART (HR: 0.69; Table 2). Factors significantly associated with an increased risk of death included age more than 30 years, an HIV transmission route other than sexual transmission, a baseline CD4+ cell count less than 100 cells/µL, a BMI less than 18.5 kg/m^2^ and WHO clinical disease stage 2 or higher. These associations remained significant in the multivariate analysis after adjusting for all potential confounders, except age in the range 30 to 39 years and a history of tuberculosis. In particular, cotrimoxazole prophylaxis was still significantly associated with a decreased risk of death (HR: 0.63).

**Table 2:** Factors associated with death in HIV-infected patients receiving antiretroviral therapy, China, 2010–2013

Factor	Risk of death	
	Unadjusted HR (95% CI)	Adjusted HR (95% CI)
**Cotrimoxazole use^a^**		
No	Reference	Reference
Yes	0.80 (0.74–0.86)	0.63 (0.56–0.70)
**Age, years**		
< 30	Reference	Reference
30–39	1.22 (1.07–1.41)	1.14 (0.99–1.32)
40–49	1.40 (1.21–1.62)	1.36 (1.17–1.57)
50–59	1.51 (1.27–1.79)	1.69 (1.42–2.01)
≥ 60	2.03 (1.69–2.45)	2.42 (1.99–2.93)
**Sex**		
Male	Reference	Reference
Female	0.75 (0.69–0.82)	0.83 (0.76–0.91)
**Marital status**		
Married or living with partner	Reference	Reference
Single, divorced or widowed	1.05 (0.97–1.13)	ND
**Route of HIV transmission**		
Sexual transmission	Reference	Reference
Blood or plasma transfusion	1.35 (1.17–1.57)	1.45 (1.25–1.70)
Injection-drug use	1.72 (1.51–1.96)	2.17 (1.88–2.50)
Other or unknown	1.19 (0.97–1.47)	1.19 (0.96–1.48)
**Baseline CD4+ cell count, cells/µL**		
100–199	Reference	Reference
50–99	2.65 (2.34–3.00)	2.52 (2.22–2.86)
< 50	4.41 (3.86–5.04)	4.23 (3.68–4.87)
**Body mass index, kg/m^2^**		
≥ 18.5	Reference	Reference
< 18.5	2.63 (2.37–2.93)	2.13 (1.90–2.39)
**WHO clinical stage**		
1	Reference	Reference
2	1.44 (1.27–1.63)	1.17 (1.03–1.33)
3	2.08 (1.82–2.36)	1.37 (1.19–1.57)
4	2.38 (2.05–2.75)	1.43 (1.22–1.67)
**Tuberculosis before ART**		
Yes	Reference	Reference
No	0.69 (0.62–0.76)	0.91 (0.82–1.01)

ART: antiretroviral therapy; CI: confidence interval; HIV: human immunodeficiency virus; HR: hazard ratio; ND: not determined; WHO: World Health Organization.

^a^ Patients were regarded as having taken cotrimoxazole if they reported drug use within 6 months of starting antiretroviral therapy. Other patients did not report cotrimoxazole use at any time.

In addition, cotrimoxazole use was associated with significantly reduced mortality over a range of ART durations: the adjusted HR for death was 0.65 for 6 months’ treatment, 0.58 for 12 months’ treatment, 0.49 for 18 months’ treatment and 0.66 for 24 months’ treatment (Table 3). There was no significant reduction in mortality for ART lasting between 25 and 30 months (HR: 0.80). The protective association of cotrimoxazole was also significant over a range of baseline CD4+ cell counts: the adjusted HR for death was 0.60 for patients with a count less than 50 cells/µL, 0.66 for those with a count of 50–99 cells/µL and 0.78 for those with a count of 100–199 cells/µL (Table 3).

**Table 3:** Cotrimoxazole and risk of death in HIV-infected patients receiving antiretroviral therapy, China, 2010–2013

Characteristic	Risk of death with cotrimoxazole versus no cotrimoxazole^a^	
	Unadjusted HR (95% CI)	Adjusted HR (95% CI)^b^
**Duration of ART, months**		
0–6	0.87 (0.79–0.96)	0.65 (0.59–0.73)
7–12	0.71 (0.60–0.85)	0.58 (0.49–0.70)
13–18	0.57 (0.44–0.72)	0.49 (0.38–0.63)
19–24	0.70 (0.52–0.95)	0.66 (0.48–0.90)
25–30	0.79 (0.50–1.24)	0.80 (0.50–1.29)
**Baseline CD4+ cell count, cells/µL**		
< 50	0.62 (0.56–0.70)	0.60 (0.54–0.67)
50–99	0.75 (0.64–0.87)	0.66 (0.56–0.78)
100–199	0.83 (0.71–0.97)	0.78 (0.62–0.98)

ART: antiretroviral therapy; CI: confidence interval; HIV: human immunodeficiency virus; HR: hazard ratio.

^a^ Patients were regarded as having used cotrimoxazole if they reported drug use within 6 months of starting antiretroviral therapy (ART). Other patients did not report cotrimoxazole use at any time.

^b^ Adjusted for age, sex, marital status, route of HIV transmission, body mass index, World Health Organization clinical stage and history of tuberculosis before ART. In addition, in calculating hazard ratios for different durations of ART, adjustment was also made for the baseline CD4+ cell count.

When the analysis was repeated with the inclusion of a category for missing data so that observations from 8753 patients who were excluded because information on their weight, height or tuberculosis status had not been reported, we found that cotrimoxazole remained significantly associated with decreased mortality (HR: 0.65; 95% CI: 0.59–0.72); this value for the HR is close to the 0.63 found in the multivariate analysis of the principle study group.

## Discussion

In this large nationally-representative study, we found that cotrimoxazole prophylaxis started early during ART reduced mortality by 37% in HIV-infected adults; the decrease was greatest among those with advanced disease. The reduction was evident as long as 24 months after ART initiation and was observed in patients with severe immunosuppression. The protective effect of cotrimoxazole has previously been documented elsewhere in ART-naïve patients.^3^^–^^12^ Our findings are consistent with those of other studies^14^^–^^20^ and reinforce Chinese national guidelines, which recommend extending the use of cotrimoxazole to patients with late-disease stage.

Although the expanded use of ART has substantially improved mortality and morbidity in HIV-infected patients around the world, several challenges remain that could undermine these gains, especially in low-resource countries. Higher mortality has been observed during the early stages of ART in both high- and low-income settings.^2^^,^^23^^,^^28^^–^^30^ In high-income settings, mortality was reported to fall from 24 per 1000 person–years during the first 6 months of treatment to 16 per 1000 person–years during months 7 to 12. In low-income settings, this trend was even more dramatic: the mortality rate was reported to fall by nearly half from 51 per 1000 person–years during the first 6 months of treatment to 27 per 1000 person–years during months 7 to 12.^2^ In sub-Saharan Africa, it has been reported that 8 to 26% of patients die during the first year of ART, with most deaths occurring in the first few months.^28^ A similar trend has been observed in China.^23^ We found that cotrimoxazole prophylaxis during the first 6 months of ART was associated with a one-third reduction in mortality. Given the high underlying risk of death in this period, this improvement in mortality could correspond to a substantial reduction in the number of deaths. Moreover, in our study, cotrimoxazole appeared to be associated with decreased mortality with a duration of ART up to about 24 months. This observation still has to be explained.

In many countries, late access to ART has been identified as an important challenge for treatment programmes.^23^^,^^24^^,^^31^^–^^34^ One study in eastern Africa found that the median baseline CD4+ cell count in patients starting ART in 2008 and 2009 was 154 cells/µL^32^ and similar findings have been reported in China.^23^^,^^24^^,^^31^ In addition, several studies have demonstrated a strong association between a low baseline CD4+ cell count and high mortality after ART initiation, especially among people with a baseline count less than 50 cells/µL.^23^^,^^24^ Although the baseline CD4+ cell count at ART initiation in China improved between 2006 and 2009, in 2009 about one third of people still started ART with a CD4+ cell count no higher than 50 cells/µL.^31^ In our study, the association between co-trimoxazole-use and reduced mortality was most marked in patients with a baseline CD4+ cell count less than 50 cells/µL: the reduction in mortality was 40%. This indicates that cotrimoxazole has the potential to reduce mortality among patients with the highest risk during their most vulnerable period.

Worldwide, the use of cotrimoxazole is suboptimal. In 2010, WHO found that, although the national policy in 38 of 41 countries surveyed was to provide cotrimoxazole to people living with HIV infections, only 25 of the 38 had fully implemented this policy.^21^ Major obstacles included an erratic drug supply, drug stocks running out and poor knowledge of cotrimoxazole among health-care workers and patients.^19^^–^^21^^,^^35^^,^^36^ In our study, we found that only half of participants received cotrimoxazole prophylaxis. Moreover, a fifth of those treated did not start the drug at ART initiation. Over the past few years, China has successfully established a working system and infrastructure for the treatment of HIV-infected patients as well as institutions for monitoring treatment – these facilities could promote more extensive use of cotrimoxazole.^22^^–^^25^

This study has several limitations. First, due to the lack of detailed information on cotrimoxazole treatment and on adherence to treatment, we equated the duration of cotrimoxazole prophylaxis to the observation time in the cotrimoxazole group. Consequently, we could not assess reduction of mortality for different exposure times of cotrimoxazole. Moreover, by regarding all patients in the cotrimoxazole group as having taken the drug continuously from the starting date, we may have underestimated the reduction in mortality. Second, data were retrieved from an observational database and may therefore have inherent biases, such as reporting or recall bias, associated with the collection of nonrandom data. However, the large number of patients in the database should have helped mitigate bias in any one direction. Finally, the large number of patients excluded because of missing data may have given rise to selection bias. However, when we repeated the analysis by including data from individuals who were excluded because details of their weight, height or history of tuberculosis were missing, our findings were consistent with those of the main analysis. Moreover, we assessed heterogeneity in baseline characteristics between included and excluded patients and found that the distributions of key variables such as age, sex and baseline CD4+ cell count were similar in the two groups. We believe, therefore, it is unlikely that our main findings were affected by biases in these key characteristics. Furthermore, our findings are consistent with those of studies conducted in other countries.^14^^–^^20^ Finally, since our data reflect the realities of treatment in a middle-income setting, our conclusions may not be generalizable to all countries.

In conclusion, by using data on a large national cohort, we found that cotrimoxazole prophylaxis administered early during ART significantly reduced mortality in HIV-infected patients, particularly in those with a CD4+ cell count less than 50 cells/µL. Given its beneficial effect on survival and its relatively low cost, cotrimoxazole should be offered in conjunction with ART to HIV-infected patients in China and other low- and middle-income countries.
